# Exacerbation of renal interstitial fibrosis by the UHRF1/G9a axis through epigenetic silencing of KLF15

**DOI:** 10.1080/0886022X.2026.2719260

**Published:** 2026-09-21

**Authors:** Jiayi Wang, Yulin Wang, Shuangxin Yuan, Xinhui Huang, Yue Yang, Yanting Shi, Yang An, Xiaobin Liu, Yulu Gu, Liang Wang, Xiaoyan Zhang

**Affiliations:** a Department of Nephrology, Zhongshan Hospital, Fudan University, Shanghai, China; b Shanghai Key Laboratory of Kidney and Blood Purification, Shanghai, China; c Shanghai Institute of Kidney and Dialysis, Shanghai, China; d Department of Nephrology, The Affiliated Wuxi People’s Hospital of Nanjing Medical University, Wuxi People’s Hospital, Wuxi Medical Center, Nanjing Medical University, Jiangsu, China; e Department of Nephrology, The Second People’s Hospital of Changzhou, the Third Affiliated Hospital of Nanjing Medical University, Jiangsu, China

**Keywords:** UHRF1, G9a, renal fibrosis, epigenetic silencing, histone modification

## Abstract

Renal fibrosis represents a central pathological driver of chronic kidney disease (CKD), yet the epigenetic mechanisms remain incompletely understood. This study identifies a critical role for the Ubiquitin-like with PHD and RING Finger domains 1 (UHRF1)/G9a axis in this process. We demonstrated that both UHRF1 and the histone methyltransferase G9a are significantly upregulated in fibrotic kidneys from CKD patients and relevant murine models by means of multiple experimental approaches, as well as in activated renal fibroblasts. Fibroblast-specific knockout of UHRF1 markedly attenuated renal fibrosis and concurrently suppressed the expression and enzymatic activity of G9a. Mechanistically, we revealed the pathological mechanism that the UHRF1/G9a axis is associated with the deposition of repressive histone H3 lysine 9 mono-/di-methylation (H3K9me1/me2) marks at the promoter of the anti-fibrotic gene Krüppel-like factor 15 (*KLF15*), as supported by chromatin immunoprecipitation-quantitative polymerase chain reaction (ChIP-qPCR) and cleavage under targets and tagmentation (CUT&Tag) assays, leading to *KLF15* transcriptional silencing. Restoration of KLF15 protein expression upon inhibition of this axis contributed to the amelioration of fibrosis. Collectively, our findings indicate the UHRF1/G9a epigenetic complex as a key regulator of renal fibrosis and highlight its potential as a novel target for alleviating renal fibrosis.

## Introduction

Chronic kidney disease (CKD) has been increasingly recognized as a major global public health burden for decades [1], with an estimated worldwide prevalence of 8%–14% [2]. Tubulointerstitial and glomerular fibroblast proliferation, particularly the progression to interstitial fibrosis, are hallmarks of CKD [3]. The activation and proliferation of these fibroblasts are closely correlated with both the severity of fibrosis and the rate of CKD progression [4]. Consequently, targeting fibroblast activation may hold promise and warrants further investigation in the context of CKD.

Ubiquitin-like with PHD and RING Finger domains 1 (UHRF1) is a multi-domain protein consisting of five functional regions: ubiquitin-like domain (UBL), tandem tudor domain (TTD), plant homeodomain (PHD), SET and RING-associated domain (SRA), and really interesting new gene domain (RING) [5]. These domains are interconnected by flexible linker regions and collectively enable UHRF1 to mediate diverse epigenetic processes, including DNA methylation, histone methylation, histone deacetylation, histone ubiquitination, and heterochromatin formation [5,6], suggesting its function as an epigenetic regulatory hub rather than merely a scaffold protein. Previous work from our group and others has established that UHRF1 plays a critical role in fibroblast activation and promotes renal fibrosis, partly through regulating DNA methylation of target genes such as Krüppel-like factor 15 (*KLF15*) [7]. Nevertheless, whether other UHRF1-dependent epigenetic mechanisms, particularly histone methylation, contribute to its pro-fibrotic function remains unexplored.

KLF15, a member of the KLF family highly expressed in the kidney, serves as a key anti-fibrotic factor [8]. It has been reported to inhibit type I collagen expression [9,10] and modulate the Wnt/β-catenin and MAPK signaling pathways, thereby suppressing the activation and proliferation of myofibroblasts [11,12].

G9a, formally designated as euchromatic histone-lysine N-methyltransferase 2 (EHMT2), is a ubiquitously expressed histone methyltransferase primarily responsible for catalyzing mono- and dimethylation of histone H3 at lysine 9 (H3K9me1/me2) [13,14]. Its functional role in promoting renal fibrosis has also been increasingly recognized [15–17]. Notably, UHRF1 has been shown to interact with G9a and form a complex that mediates H3K9 methylation, a modification associated with transcriptional repression [18–20]. However, the role of the UHRF1-G9a epigenetic regulatory complex in renal fibrosis and its downstream candidate genes remains explored.

Based on this evidence, we hypothesize that UHRF1 mediates G9a to catalyze H3K9 methylation at KLF15 promoter, resulting in its epigenetic silencing and consequent exacerbation of renal fibrosis. This study aims to investigate whether disruption of the UHRF1-G9a-KLF15 axis can alleviate renal fibrosis.

## Materials and methods

Due to space constraints, comprehensive details of all experimental methods are provided in the Supplementary Methods. Therefore, the main text describes only the key experimental protocols and analysis methods that were commonly applied across the study.

### Human renal biopsy samples

Human renal biopsy samples were acquired as part of routine clinical diagnostics from patients admitted to the department of Nephrology, Zhongshan Hospital, Fudan University between June 2021 and December 2022 (clinical details are summarized in Supplementary Table S1). All participants provided written informed consent, and the study protocol was approved by the Ethics Committee of Zhongshan Hospital, Fudan University (Approval No. B2021-346R). All procedures performed in studies involving human participants were in accordance with the 1964 Helsinki Declaration and its later amendments or comparable ethical standards. Additional methodological details regarding sample processing and characterization are provided in the Supplementary Methods.

### Animals and treatment

Male C57BL/6 mice (6–8 weeks old) were used to establish models of renal fibrosis *via* unilateral ureteral obstruction (UUO) or unilateral ischemia-reperfusion (UIR) surgery. As both models preserve a functionally intact contralateral kidney that provides full compensatory function, they effectively avoid confounding factors such as hemodynamic alterations and uremic toxin accumulation, thereby allowing us to better focus specifically on renal interstitial fibrosis itself [21,22]. Kidney tissues were collected 1 week after UUO or 2 weeks after UIR. Sham-operated mice underwent kidney exposure without ligation or ischemia. In the experiments involving treatment with the inhibitor BIX-01294, because previous studies have suggested that a single injection of BIX-01294 might not achieve a long-lasting inhibitory effect [15]; therefore, in combination with our preliminary experimental results (Figure S1), we administered BIX-01294 *via* daily intraperitoneal injection at a dose of 2 mg/kg, beginning one day after surgery and continuing until tissue collection.

All mouse procedures, including housing, modeling, anesthesia (*via* intraperitoneal injection of 2% sodium pentobarbital at 50 mg/kg), and euthanasia method (cervical dislocation in accordance with the American Veterinary Medical Association guidelines), have been reviewed and approved by the Institutional Animal Care and Use Committee of Fudan University (Approval number: 202312008S). For Western blotting, immunohistochemistry, immunofluorescence, and Masson’s trichrome staining, six mice per group were used for the main experiments, whereas three mice per group were used in the preliminary experiments for pilot exploration (Figure S1). For ChIP-qPCR assays, three mice per group were used throughout. Detailed animal procedures are provided in the Supplementary Materials.

### Cell culture

The rat normal renal fibroblast line NRK-49F was maintained in high-glucose Dulbecco’s Modified Eagle Medium (DMEM) supplemented with 10% fetal bovine serum (FBS) and 1% penicillin-streptomycin. For NRK-49F cell activation experiments, cells were stimulated with 10 ng/mL TGF-β1 for 48 h. For experiments involving BIX-01294 treatment, cells were incubated with 2.5 μM BIX-01294 for 48 h after activation. Detailed cell culture procedures are described in the Supplementary Methods.

### Public database analysis

For each dataset, we selected only specific groups of interest for our analysis. The public databases we included were GSE66494 (comparing kidney transcriptomic data from 53 CKD patients and 8 healthy controls), GSE269356 (data comparing TGF-β1-stimulated NRK-49F cells at 5 ng/mL for 24 h with untreated controls), GSE299417 (data comparing the WT mouse Sham + vehicle group and the UUO 14d + vehicle group), and GSE293589 (data comparing the WT Sham group and the UIR 14d group). All datasets were retrieved from the Gene Expression Omnibus (GEO) database (https://www.ncbi.nlm.nih.gov/geo/). For datasets with raw count data, differential expression analysis was performed using the DESeq2 package (v1.34.0) in R (v4.1.0). For datasets with FPKM-normalized expression values, differential expression analysis was performed using the limma package (v3.50.0) with the voom function. Genes with an |log_2_FC| > 1 and an adjusted.*p* < 0.05 were considered significantly differentially expressed. Volcano plots were generated using the ggplot2 package (v3.4.0) in R (v4.1.0) to visualize the distribution of differentially expressed genes (DEGs).

### Quantification and statistical analysis

All data are presented as mean ± standard error of the mean (SEM). For comparisons between two groups, an unpaired two-tailed Student’s *t*-test was used. For comparisons involving a single independent variable with three or more groups, one-way analysis of variance (ANOVA) was performed, followed by Tukey’s *post hoc* test for multiple comparisons. For multiple comparisons involving two independent variables, two-way ANOVA was used, followed by Šidák’s *post hoc* test as appropriate. All statistical tests were two-tailed, and a *p-*value < 0.05 was considered statistically significant. Analyses were conducted using GraphPad Prism version 9.1.1.

## Results

### Co-upregulation of UHRF1 and G9a correlates with severity of chronic kidney disease

**Figure 1.**
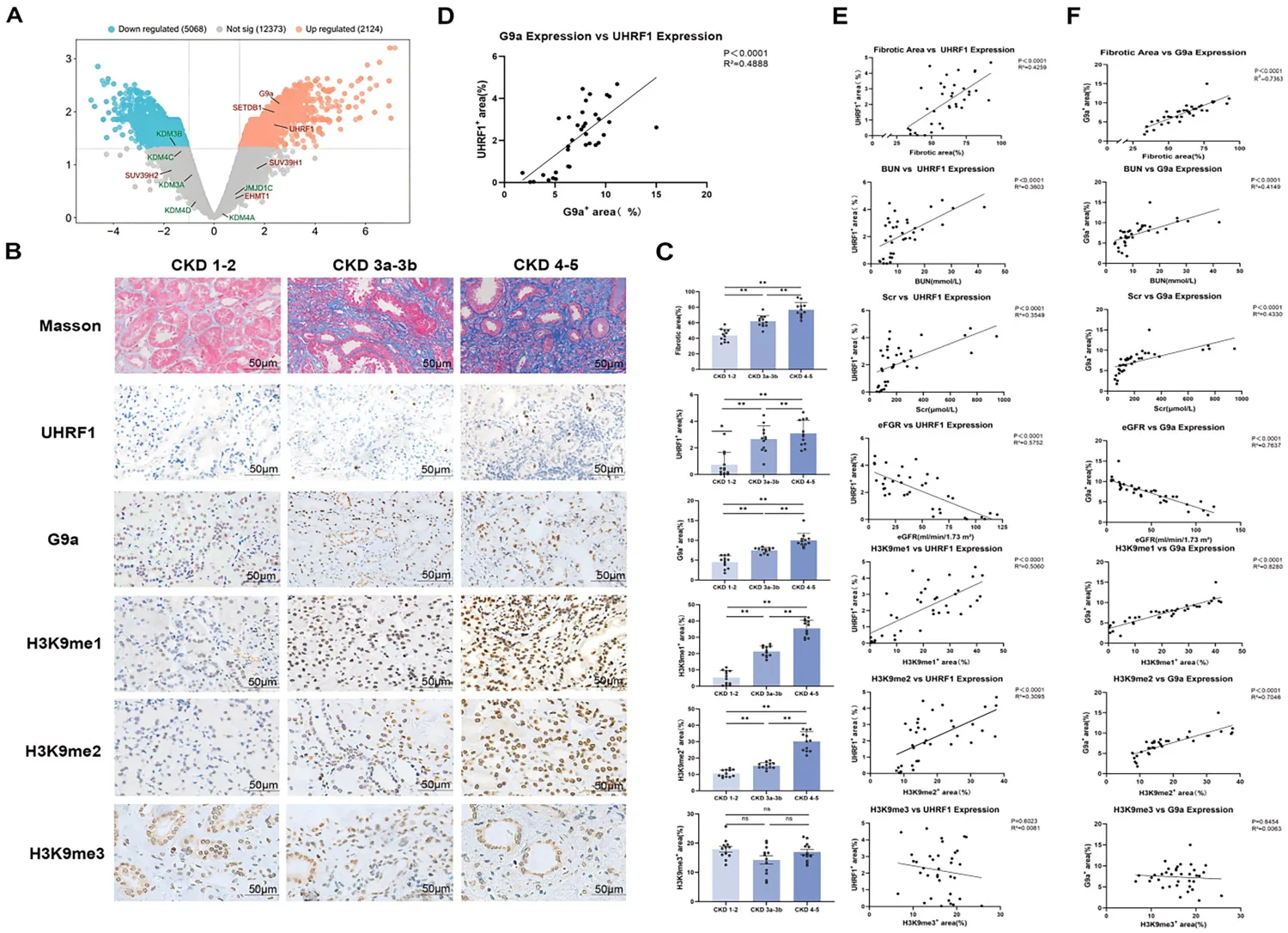
Co-upregulation of UHRF1 and G9a correlates with severity of chronic kidney disease. (A) Transcript levels of *UHRF1* and key H3K9 methyltransferases/demethylases in renal tissues from CKD patients and healthy controls (GSE66494 dataset). Differentially expressed genes were identified using the DESeq2 package (v1.34.0) with an adjusted.*p* < 0.05 and |log_2_FC| > 1. (B) Representative images of Masson’s trichrome staining and immunohistochemistry for UHRF1, G9a, H3K9me1, H3K9me2, and H3K9me3 in kidney sections from patients across CKD stages 1–5 (scale bar: 50 μm). **C** Quantification of fibrotic area and positive staining areas for UHRF1, G9a, H3K9me1, H3K9me2, and H3K9me3 across CKD stages. (D) Correlation between UHRF1 and G9a staining intensity. (E and F) Spearman correlation analysis of UHRF1 (E) and G9a (F) staining intensity with fibrotic area, clinical parameters (Scr, BUN, eGFR), and histone marks (H3K9me1, H3K9me2, H3K9me3). Data are presented as mean ± SEM (*n* = 6 patients per stage). For group comparisons, adjacent stages were pooled into three groups (*n* = 12 patients each). Statistical significance was assessed by one-way ANOVA with Tukey’s post-hoc test or Spearman correlation. *p < 0.05, **p < 0.01.

Transcriptional analysis of the public dataset GSE66494 (53 CKD patients *vs.* 8 healthy controls) revealed that mRNA levels of *UHRF1* and *G9a* were significantly upregulated in renal tissues from patients with CKD compared to healthy controls (Figure 1A). Among other key methyltransferases and demethylases regulating methylation of histone H3 at lysine 9 (H3K9), including suppressor of variegation 3–9 homolog 1 (*SUV39H1*), suppressor of variegation 3–9 homolog 2 (*SUV39H2*), and SET domain bifurcated histone lysine methyltransferase 1 (*SETDB1*), *EHMT1* (euchromatic histone-lysine N-methyltransferase) and members of the lysine-specific demethylases (KDM) 3 and 4 families, only *SETDB1* showed a concurrent significant increase (Figure 1A).

To validate these findings, we enrolled a total of 36 CKD patients from the Department of Nephrology, Zhongshan Hospital, Fudan University (Ethics Approval No. B2021-346R), with 6 patients included at each of the CKD stages 1, 2, 3a, 3b, 4, and 5. For analysis, adjacent stages were combined into three groups of 12 patients each. Clinical parameters, including serum creatinine (Scr), blood urea nitrogen (BUN), and estimated glomerular filtration rate (eGFR), were collected. Renal biopsy tissues were subjected to pathological evaluation. Immunohistochemical analysis showed that patients with more severe CKD stages had higher renal expression of UHRF1 and G9a, along with elevated global renal expression of H3K9me1 and H3K9me2. However, no significant changes were observed for H3K9me3 or SETDB1 across different CKD stages (Figures 1B, C and S^2^).

Correlation analyses were performed among UHRF1, G9a, SETDB1, histone modification markers (H3K9me1/me2/3), and clinical parameters (Scr, BUN, eGFR, and Masson-stained fibrotic area). A strong positive correlation was observed between UHRF1 and G9a expression levels (Figure 1D). Additionally, UHRF1 and G9a expression levels each showed significant positive correlations with Scr, BUN, fibrotic area, H3K9me1, and H3K9me2, while correlating negatively with eGFR (Figure 1E and F); however, neither showed a significant correlation with H3K9me3 expression. Notably, SETDB1 did not exhibit significant correlations with any of the variables (Figure S2). SETDB1 is mainly responsible for H3K9me3 modification of histones [23]. The comparable SETDB1 expression across CKD stages could partially explain the non-significant H3K9me3 levels in the kidney. Based on the expression pattern across disease severity strata, and significant correlations with fibrosis severity and specific histone marks, UHRF1 and G9a were selected as the primary focus for subsequent mechanistic investigations.

### The UHRF1/G9a epigenetic axis is upregulated in experimental renal fibrosis and TGF-β1-stimulated fibroblasts

**Figure 2.**
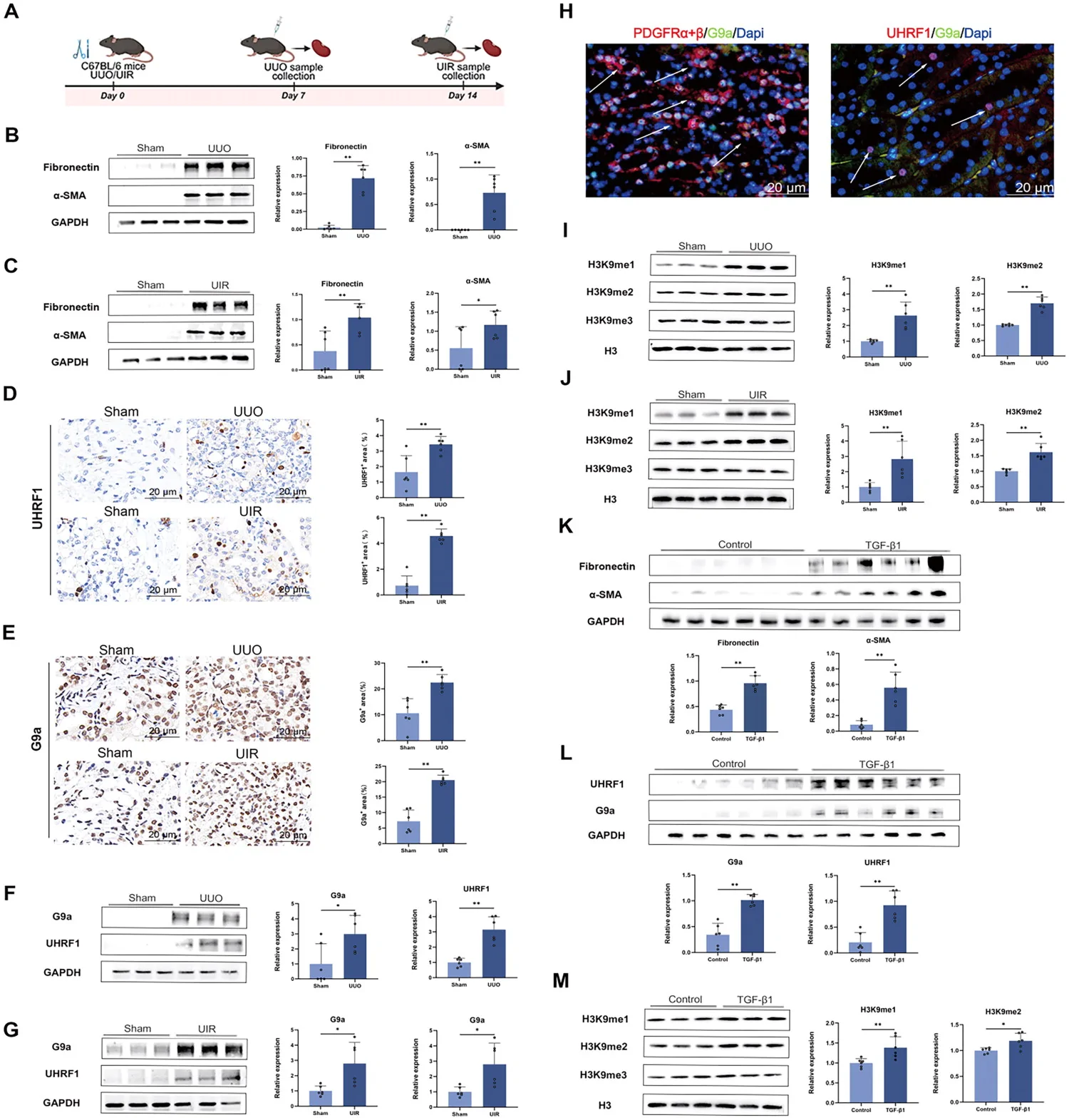
The UHRF1/G9a epigenetic axis is upregulated in experimental renal fibrosis and TGF-β1-stimulated fibroblasts. (A) Schematic of the UUO and UIR mouse models. (B and C) Western blot analysis of fibronectin and α-SMA in kidney tissues from sham, UUO, and UIR mice (*n* = 6 mice per group). (D and E) Immunohistochemical quantification of UHRF1^+^ and G9a^+^ areas in renal tissues (*n* = 6 mice per group). (F and G) Upregulation of UHRF1 and G9a protein levels in fibrotic kidneys from UUO and UIR models (*n* = 6 mice per group). (H) Representative immunofluorescence images showing co-localization of G9a with PDGFRα + β^+^ cells and UHRF1 in fibrotic kidneys (arrows indicate co-localization; scale bar: 50 μm). (I and J) Western blot analysis of H3K9me1, H3K9me2, and H3K9me3 levels in sham and fibrotic kidneys (*n* = 6 mice per group). (K and L) Upregulation of fibronectin, α-SMA, UHRF1, and G9a in NRK-49F renal fibroblasts stimulated with TGF-β1 (*n* = 3 independent cell experiments). (M) Increased H3K9me1 and H3K9me2 levels in TGF-β1-treated NRK-49F cells (*n* = 3 independent cell experiments). Data are presented as mean ± SEM. Statistical analysis was performed using an unpaired *t*-test between two groups. *p < 0.05, **p < 0.01.

To investigate the role of UHRF1 and G9a in renal fibrosis, we utilized two well-established murine models: UUO for 7 days and UIR for 14 days (Figure 2A).

To date, no consensus has been reached regarding a specific fibroblast marker [24]. However, α-smooth muscle actin (α-SMA) is still commonly used to mark myofibroblasts [25,26]. Moreover, fibronectin is reported as the main component of extracellular matrix (ECM), and thus it’s often used as a surrogate marker for renal interstitial fibrosis [27]. Although PDGFRα and PDGFRβ mark not only fibroblasts but also other mesenchymal cells such as pericytes and vascular smooth muscle cells, they remain valuable markers for identifying fibroblast-lineage cells, particularly when used in combination in the fibrotic kidney, as some studies have identified these PDGFRα + β^+^ cells as a major cellular source of ECM in the fibrotic human kidney [28,29]. Therefore, in this study, we used the α-SMA^+^ area, fibronectin^+^ area and PDGFRα + β^+^ cell population as the strategy to assess renal fibrosis. Additionally, to distinguish proximal tubules, we used AQP1 as a marker to identify proximal renal tubules [30].

In both models, renal fibrosis was confirmed by a significant upregulation of α-SMA^+^ area, fibronectin^+^ area, and PDGFRα + β^+^ cell proportion (Figure 2B and C; Figure S3A). Consistently, the protein expression of both UHRF1 and G9a was markedly increased in fibrotic kidneys compared to sham-operated controls (Figure 2D–G; Figure S3B). Immunofluorescence analysis revealed that G9a was co-localized with PDGFRα + β^+^ cells as well as AQP1^+^ cells, reflecting its widespread expression in kidneys (Figure 2H, Figure S3B). However, UHRF1 was primarily localized in PDGFRα + β^+^ cells. Furthermore, G9a showed clear co-localization with UHRF1 (Figure 2H, Figure S3B). Mirroring the observations in human CKD tissues, the levels of H3K9me1 and H3K9me2, but not H3K9me3, were significantly elevated in the fibrotic murine kidneys (Figure 2I, J).

We further validated these findings *in vitro* using the renal fibroblast line NRK-49F. Treatment with transforming growth factor-β1 (TGF-β1), a key profibrotic cytokine, could induce the expression of α-SMA and fibronectin, indicating the activation of NRK-49F cells (Figure 2K). Based on literature review and preliminary experiments, NRK-49F cells were treated with 10 ng/mL TGF-β1 for 48 h as the intervention condition (Figure S4A and B). Concomitantly, TGF-β1 stimulation led to a significant upregulation of UHRF1 and G9a proteins (Figure 2L), as well as increased global levels of H3K9me1 and H3K9me2 (Figure 2M). These results confirm that the upregulation of this epigenetic axis is a consistent feature of fibroblast activation across species and experimental settings.

### UHRF1 regulates the expression and enzymatic activity of G9a in renal fibrosis

**Figure 3.**
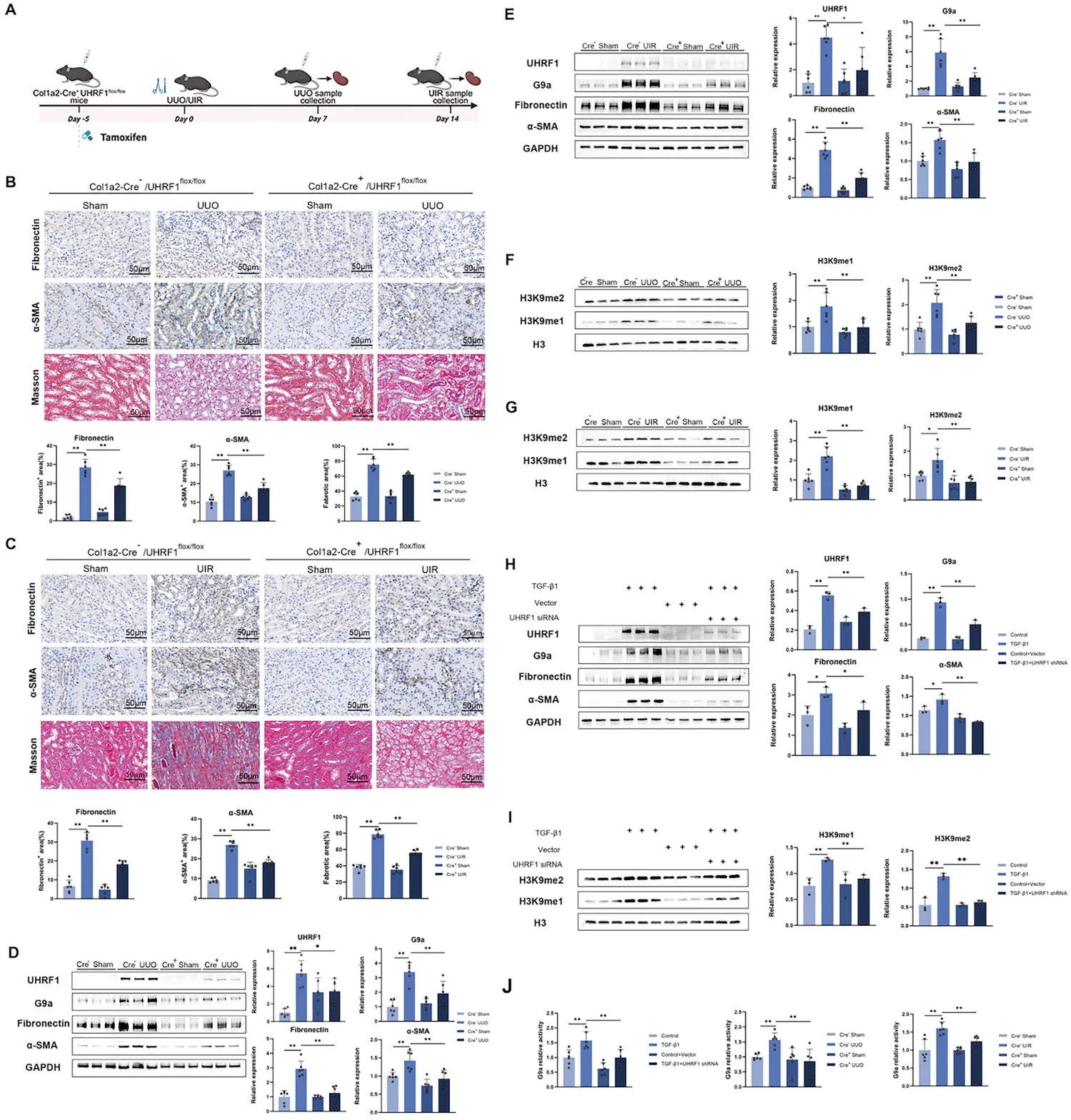
UHRF1 regulates the expression and enzymatic activity of G9a in renal fibrosis. (A) Schematic of fibroblast-specific *UHRF1* knockout (Col1a2-Cre^+^ *UHRF1*^flox/flox^) in UUO and UIR mouse models. (B and C) Representative images and quantification of fibronectin and α-SMA immunohistochemistry, and Masson’s trichrome staining, showing attenuated fibrosis following *UHRF1* deletion (scale bar: 50 µm; *n* = 6 mice per group). (D and E) Western blot analysis of UHRF1, G9a, and fibrotic markers (α-SMA, fibronectin) in kidneys from control and *UHRF1* knockout mice subjected to UUO or UIR injury (*n* = 6 mice per group). (F and G) Reduction of H3K9me1 and H3K9me2 levels in fibrotic kidneys upon *UHRF1* knockout (*n* = 6 mice per group). (H and I) *In vitro* validation in NRK-49F cells: siRNA-mediated *UHRF1* knockdown downregulates G9a protein expression, suppresses TGF-β1-induced fibrotic markers, and reduces H3K9me1/me2 (*n* = 3 independent cell experiments). (J) Decrease in G9a enzymatic activity *in vitro* and *in vivo* following *UHRF1* depletion. Data are presented as mean ± SEM. Statistical significance was determined by two-way ANOVA followed by Šidák’s *post hoc* test. *p < 0.05, **p < 0.01.

To determine whether UHRF1 regulates G9a *in vivo*, we generated mice with fibroblast-specific deletion of *UHRF1* by crossing *UHRF1*^flox/flox^ mice with Col1a2-Cre/ERT2 mice (Col1a2-Cre^+^ *UHRF1*^flox/flox^), as previously described [7]. Following tamoxifen-induced knockout, renal fibrosis was induced *via* UUO or UIR (Figure 3A). Fibroblast-specific ablation of *UHRF1* markedly attenuated renal fibrosis in both UUO and UIR models, as evidenced by the decreased fibrotic area and the reduced expression of α-SMA and fibronectin (Figure 3B–E). However, we found that neither the UUO nor the UIR model induced significant alterations in serum creatinine levels in mice, likely because the contralateral healthy kidney remained fully compensatory within the observation period (Figure S5A and B). Accordingly, fibroblast-specific *UHRF1* knockout did not significantly affect renal function parameters under these experimental conditions (Figure S5A and B). Concomitantly, G9a protein levels were significantly decreased in the knockout mice (Figure 3D, E). The increases in H3K9me1 and H3K9me2 observed in fibrotic control kidneys were substantially reversed upon *UHRF1* deletion (Figure 3F and G). Consistent with the *in vivo* findings, siRNA-mediated knockdown of *UHRF1* in TGF-β1-stimulated NRK-49F fibroblasts led to a parallel downregulation of G9a protein and global H3K9me1/me2 level (Figure 3H and I). Importantly, following *UHRF1* silencing, the relative enzymatic activity of G9a was also significantly reduced after being normalized to its protein expression levels (Figure 3J). These results demonstrate that UHRF1 is necessary for maintaining both the expression and functional activity of G9a in the context of renal fibrosis.

### The SRA and RING domains of UHRF1 are critical for its interaction with G9a

**Figure 4.**
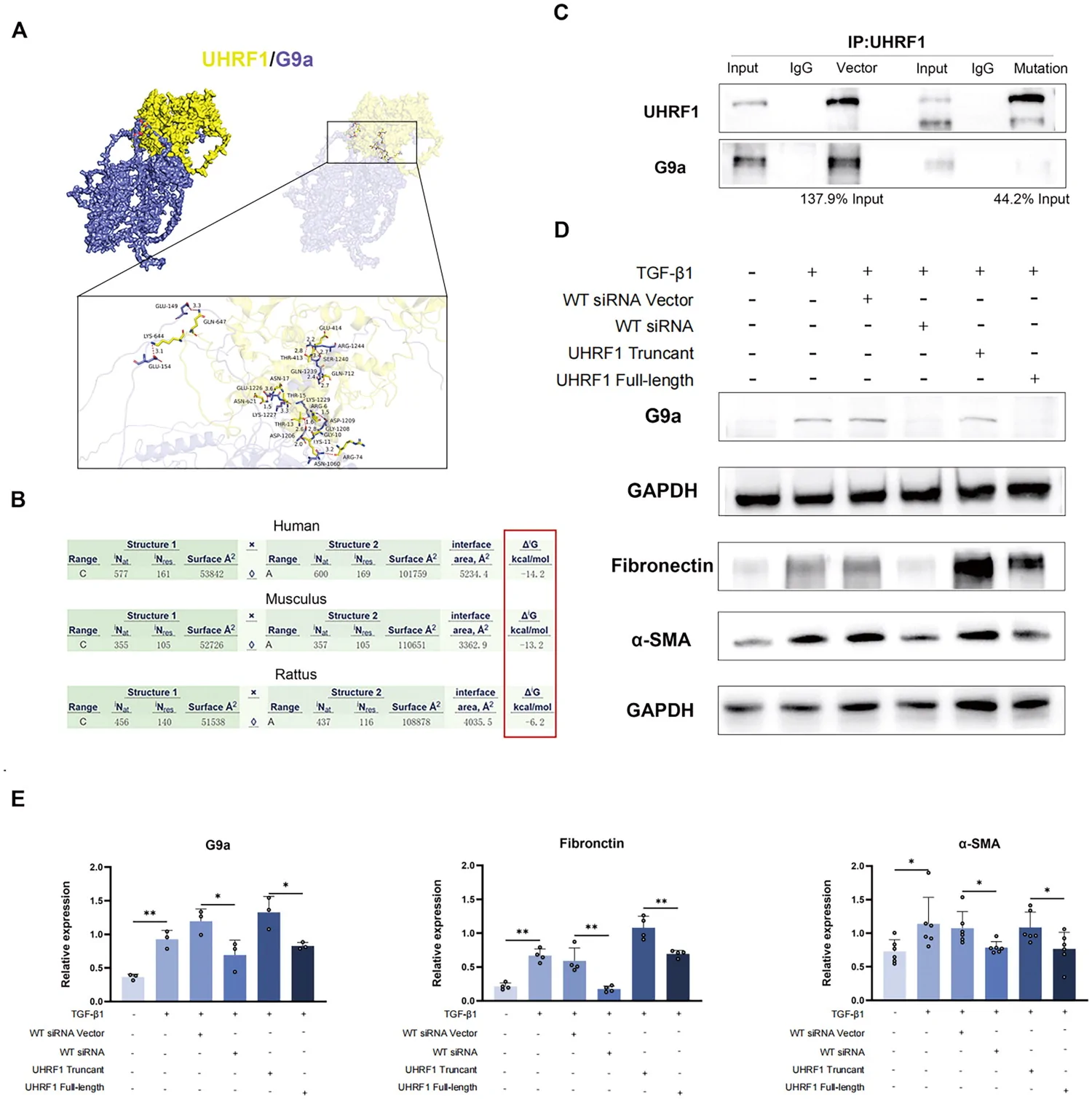
The SRA and RING domains of UHRF1 are critical for its interaction with G9a. (A) Structural modeling of the UHRF1-G9a protein-protein interaction in humans, simulated using GRAMM-X (https://swift.cmbi.umcn.nl/servers/html/model.html). (B) Predicted binding modes and calculated binding free energies for the UHRF1-G9a complex across human, mouse, and rat orthologs (GROMACS 2025.3). (C) Co-immunoprecipitation assay showing that the amount of G9a co-precipitated with the UHRF1 truncation mutant was markedly reduced compared with that co-precipitated with full-length UHRF1. (D and E) Expression of G9a and fibrotic markers in cells transduced with UHRF1 truncation mutant were significantly downregulated compared with full-length UHRF1. Data are presented as mean ± SEM (*n* = 3 for G9a, *n* = 4 fibronectin and *n* = 6 for α-SMA). Statistical significance was determined by an unpaired *t*-test between two adjacent groups. *p < 0.05, **p < 0.01.

To characterize the molecular interaction between UHRF1 and G9a, we performed molecular docking and molecular dynamics simulations using protein structures from humans, mice and rats (Figure 4A, Figure S6A and B). The calculated binding free energies for the UHRF1-G9a complex were −14.2 kcal/mol (human), −13.2 kcal/mol (mouse), and −6.2 kcal/mol (rat) (Figure 4B, Supplemental Table S2). Since a binding free energy below −5 kcal/mol is indicative of a stable interaction [31], these computational predictions suggest a relatively robust and conserved binding affinity across species.

Using the human complex as a representative model, docking analysis predicted that the SRA and RING domains of UHRF1 are the primary sites mediating the interaction with G9a (Figure 4A), which aligns with prior structural reports [19]. We generated truncated *UHRF1* mutants with deletion of the SRA and RING domains, packaged them into lentiviral vectors, and transduced them into NRK-49F cells. Co-immunoprecipitation (Co-IP) analysis revealed that the binding ability between UHRF1 and G9a in the lentivirus-transfected truncation mutant group was significantly decreased compared with the full-length UHRF1 group (137.9% of input *vs.* 44.2% of input). Successful expression of the truncation mutants was confirmed by a visible shift in molecular weight on Western blot, with a reduced band at ∼97 kDa and a concomitant stronger band at ∼70 kDa (Figure 4C). Furthermore, functional validation showed that TGF-β1-stimulated NRK-49F cells infected with these truncation mutants exhibited decreased G9a expression, accompanied by a significant reduction in the levels of fibrotic markers compared to vector controls of full-length UHRF1 (Figure 4D and E). These findings demonstrate that UHRF1 and G9a associate within a protein complex, and that the SRA and RING domains of UHRF1 are essential for this association and its downstream functional effects.

### G9a serves as a critical downstream effector through which UHRF1 mediates renal fibrosis

**Figure 5.**
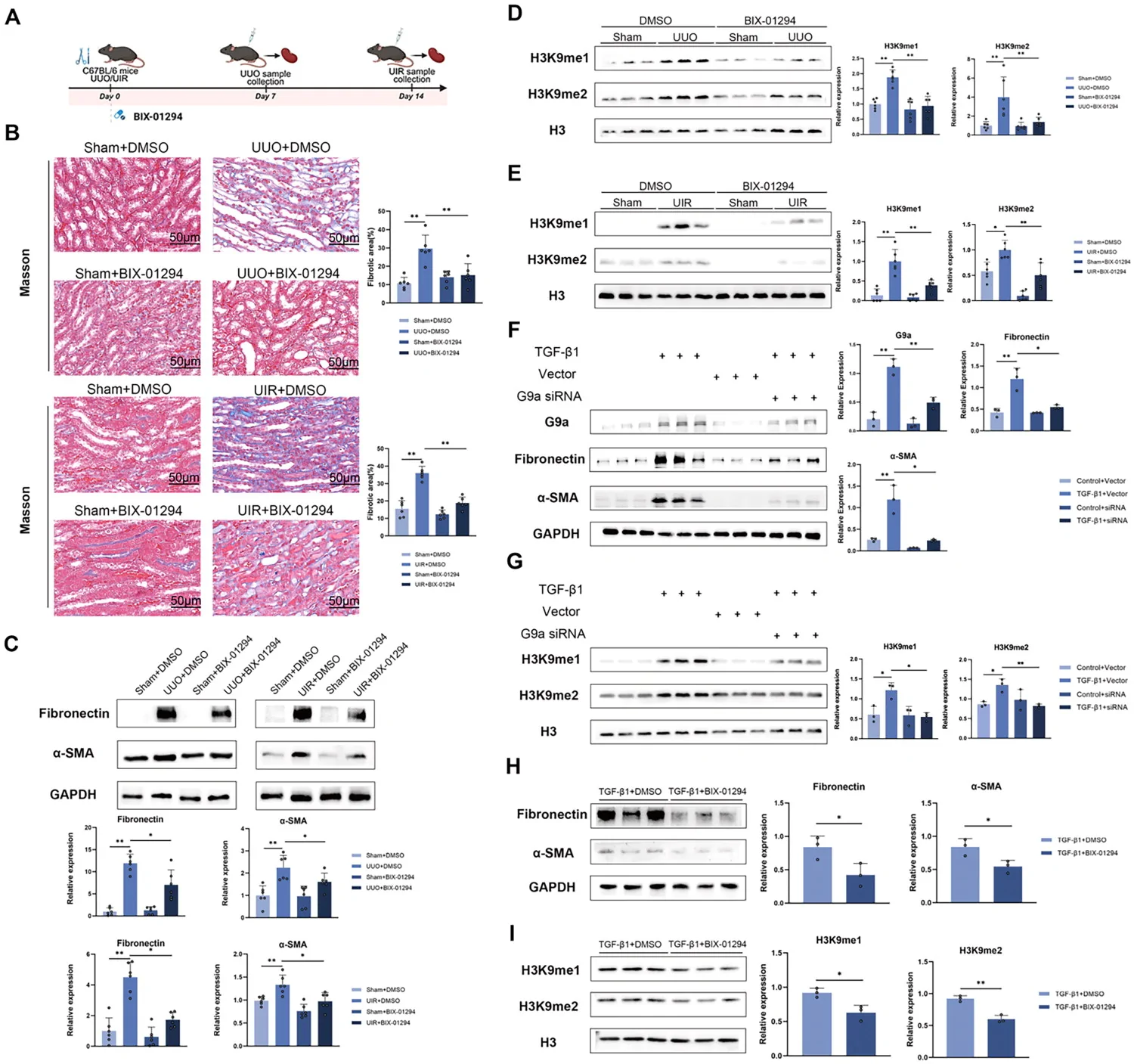
G9a serves as a critical downstream effector through which UHRF1 mediates renal fibrosis. Mice subjected to UUO or UIR injury received daily intraperitoneal injections of the G9a inhibitor BIX-01294 (2 mg/kg). (B) Representative Masson’s trichrome staining and quantification of fibrotic area, demonstrating amelioration of renal fibrosis upon G9a inhibition (scale bar: 50 µm; *n* = 6 mice per group). **C** Western blot analysis showing reduced expression of fibronectin and α-SMA in BIX-01294 treated fibrotic kidneys (*n* = 6 mice per group). (D and E) Reduction of H3K9me1 and H3K9me2 levels in fibrotic kidneys following BIX-01294 treatment (*n* = 6 mice per group). (F) *In vitro* knockdown of *G9a via* siRNA in NRK-49F cells suppresses TGF-β1-induced fibrotic marker expression and global H3K9me1/me2 levels (*n* = 3 independent cell experiments). (G) Pharmacological inhibition of G9a activity with BIX-01294 similarly attenuates fibrotic marker expression in activated NRK-49F cells (*n* = 3 independent cell experiments). (H) Western blot analysis showing reduced expression of fibronectin and α-SMA in BIX-01294-treated NRK-49F cells (*n* = 3 independent cell experiments). (I) Reduction of H3K9me1 and H3K9me2 levels in TGF-β1 induced NRK-49F cells following BIX-01294 treatment (*n* = 3 independent cell experiments). Data are presented as mean ± SEM. Statistical significance was determined using unpaired two-tailed *t*-test for two-group comparisons, and two-way ANOVA with Šidák’s *post hoc* test for multiple-group comparisons. *p < 0.05, **p < 0.01.

Having established that UHRF1 regulates G9a to promote renal fibrosis, we next investigated whether G9a itself contributes to fibrogenesis independently of UHRF1. To address this, we inhibited G9a pharmacologically *via* BIX-01294, a competitive inhibitor that binds the substrate pocket of G9a, thereby specifically reducing H3K9me1 and H3K9me2 levels [32,33]. BIX-01294 was administered intraperitoneally (2 mg/kg/day) to mice subjected to UUO or UIR injury (Figure 5A). BIX-01294 treatment markedly attenuated renal fibrosis in both models, as shown by decreased fibrotic area and decreased expression of α-SMA and fibronectin (Figure 5B and C). This therapeutic effect was accompanied by significant decreases in global H3K9me1 and H3K9me2 (Figure 5D and E).

The drug concentration of BIX-01294 (2.5 μM) used *in vitro* studies was determined by a CCK-8 assay, which confirmed it did not induce significant cytotoxicity (Figure S7). Consistent with the *in vivo* findings, siRNA-mediated knockdown of G9a or its pharmacological inhibition with BIX-01294 in TGF-β1-stimulated NRK-49F cells significantly suppressed fibroblast activation and reduced H3K9me1/me2 levels (Figure 5F-I).

Moreover, the expression of UHRF1 was not altered by BIX-01294 treatment (Figure S8A and B), suggesting that the anti-fibrotic effect of G9a inhibition is not mediated by downregulation of UHRF1 protein expression. These observations raise the possibility that G9a may function downstream of UHRF1. To further validate our hypothesis, we found that G9a overexpression partially increased the protein expression of activation markers in UHRF1-knockdown activated fibroblasts (Figure S9A). Thus, we propose that G9a acts downstream of UHRF1 and partially contributes to the promotion of renal fibrosis.

### UHRF1-G9a promotes H3K9 methylation of the *KLF15* promoter and transcriptional repression of *KLF15*

**Figure 6.**
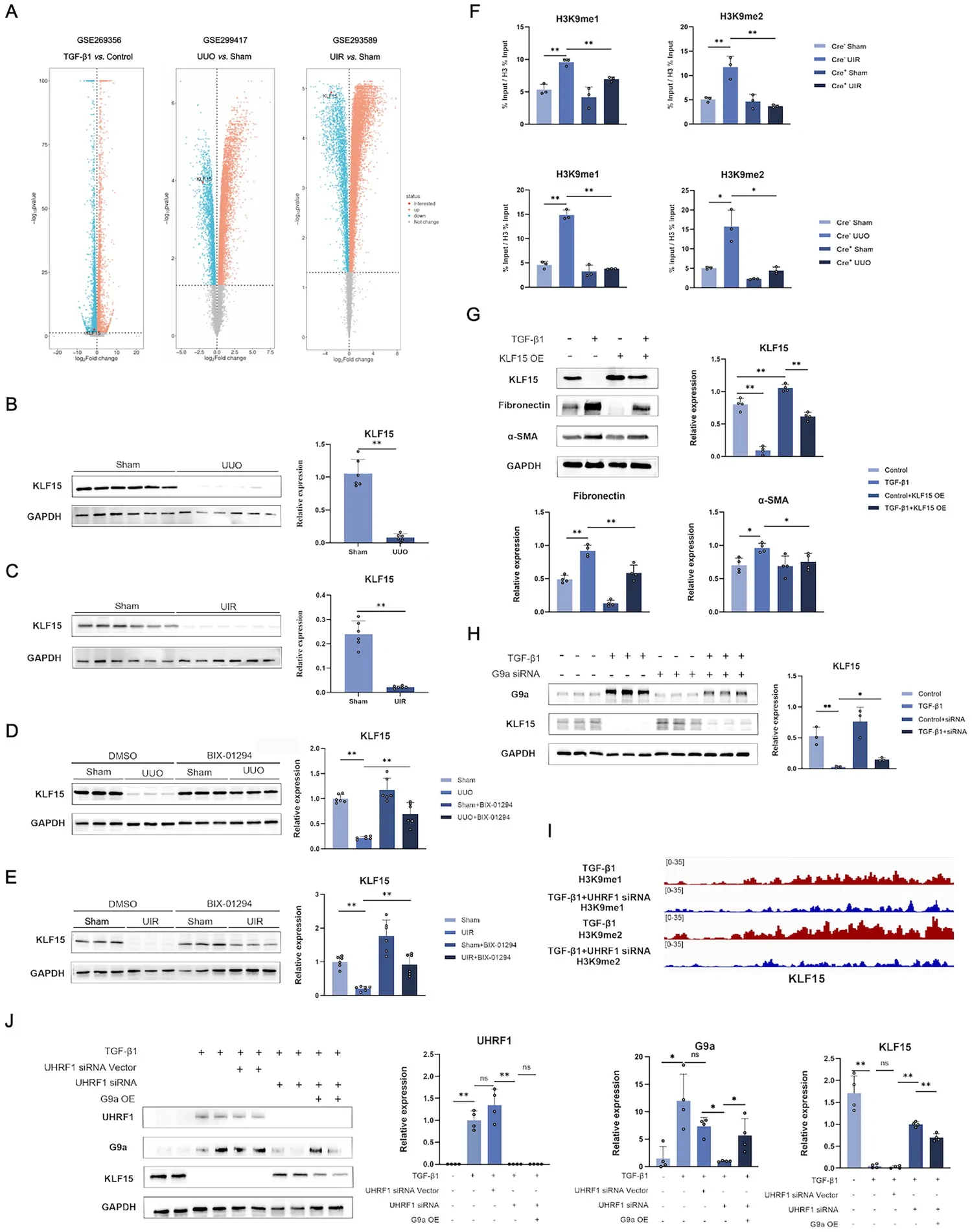
UHRF1-G9a promotes H3K9 methylation of the *KLF15* promoter and transcriptional repression of *KLF15*. (A) Volcano plots highlighting *KLF15* as significantly downregulated in activated NRK-49F cells (GSE269356), kidneys 14 days post-UUO surgery (GSE299417) and kidneys 14 days post-UIR (GSE293589). (B and C) KLF15 protein expression is suppressed in UUO and UIR kidneys (*n* = 6 mice per group). (D and E) Pharmacological inhibition of G9a with BIX-01294 partially restores KLF15 protein levels in fibrotic kidneys (*n* = 6 mice per group). (F) ChIP-qPCR shows increased H3K9me1 and H3K9me2 enrichment at the *KLF15* promoter in fibrotic kidneys, which is reversed upon fibroblast-specific UHRF1 knockout (*n* = 3 mice per group). (G) Overexpression of *KLF15* suppresses TGF-β1 induced fibrotic marker expression (*n* = 3 independent cell experiments). (H) *G9a* knockdown prevents TGF-β1 mediated downregulation of KLF15 protein expression (*n* = 3 independent cell experiments). (I) CUT&Tag analysis confirms that *UHRF1* knockdown reduces H3K9me2 enrichment at the *KLF15* promoter in activated fibroblasts. **J** Rescue experiment: *UHRF1* knockdown upregulates KLF15 expression, while co-overexpression of *G9a* abolishes this effect, without significantly altering UHRF1 levels (*n* = 4 independent cell experiments). Data are presented as mean ± SEM. Statistical significance was determined using unpaired two-tailed *t*-test for two-group comparisons, and two-way ANOVA with Šidák’s *post hoc* test for multiple-group comparisons. *p < 0.05, **p < 0.01.

Although a large number of DEGs were observed across various kidney fibrosis models following injury, downregulation of KLF15 at the transcriptional level was observed in three distinct fibrotic models (Figure 6A). Currently, several studies have reported that KLF15 possesses potential anti-fibrotic and renoprotective effects [34,35]. Therefore, we sought to explore whether *KLF15* might serve as a downstream candidate gene through which UHRF1-G9a exerts its epigenetic regulatory effects to counteract renal fibrosis.

First, we confirmed that KLF15 protein expression was suppressed in fibrotic mouse kidneys (Figure 6B and C), and this suppression was partially reversed by treatment with the G9a inhibitor BIX-01294 in both UUO and UIR models (Figure 6D and E). Given the repressive role of H3K9me1/me2, we performed ChIP-qPCR to determine whether the downregulation of KLF15 in fibrotic kidneys was associated with enrichment of these marks at its promoter. Indeed, we observed significant enrichment of H3K9me1/me2 at *KLF15* promoter in fibrotic kidneys, which was markedly reduced upon fibroblast-specific *UHRF1* knockout (Figure 6F).

*In vitro*, TGF-β1 activation of NRK-49F cells downregulated KLF15 protein expression and overexpression of *KLF15* in these cells could partially reverse the TGF-β1-induced upregulation of fibrotic markers (Figure 6G). Conversely, knockdown of *G9a* prevented TGF-β1-induced KLF15 protein downregulation (Figure 6H). Using CUT&Tag, we further demonstrated that *UHRF1* knockdown reduced H3K9me1/me2 enrichment at *KLF15* promoter in activated fibroblasts (Figure 6I).

Finally, a rescue experiment further suggested G9a as the key mediator of UHRF1 action on KLF15. While *UHRF1* siRNA increased KLF15 protein expression, co-transfection with a *G9a* overexpression plasmid partially abolished this effect (Figure 6J). Notably, *G9a* overexpression did not alter UHRF1 protein levels, further supporting its role as a downstream effector in this regulatory axis (Figure 6J). In ChIP-qPCR assays, we observed that *UHRF1* siRNA reduced the enrichment of H3K9me1/me2 at the *KLF15* promoter region in activated fibroblasts. Furthermore, *G9a* overexpression following *UHRF1* knockdown led to a modest yet statistically significant increase in H3K9me1/me2 enrichment (Figure S9B). These findings collectively suggest that UHRF1 functions upstream of G9a as a scaffold protein, contributing to both renal fibrosis and *KLF15* transcriptional silencing.

## Discussion

**Figure 7.**
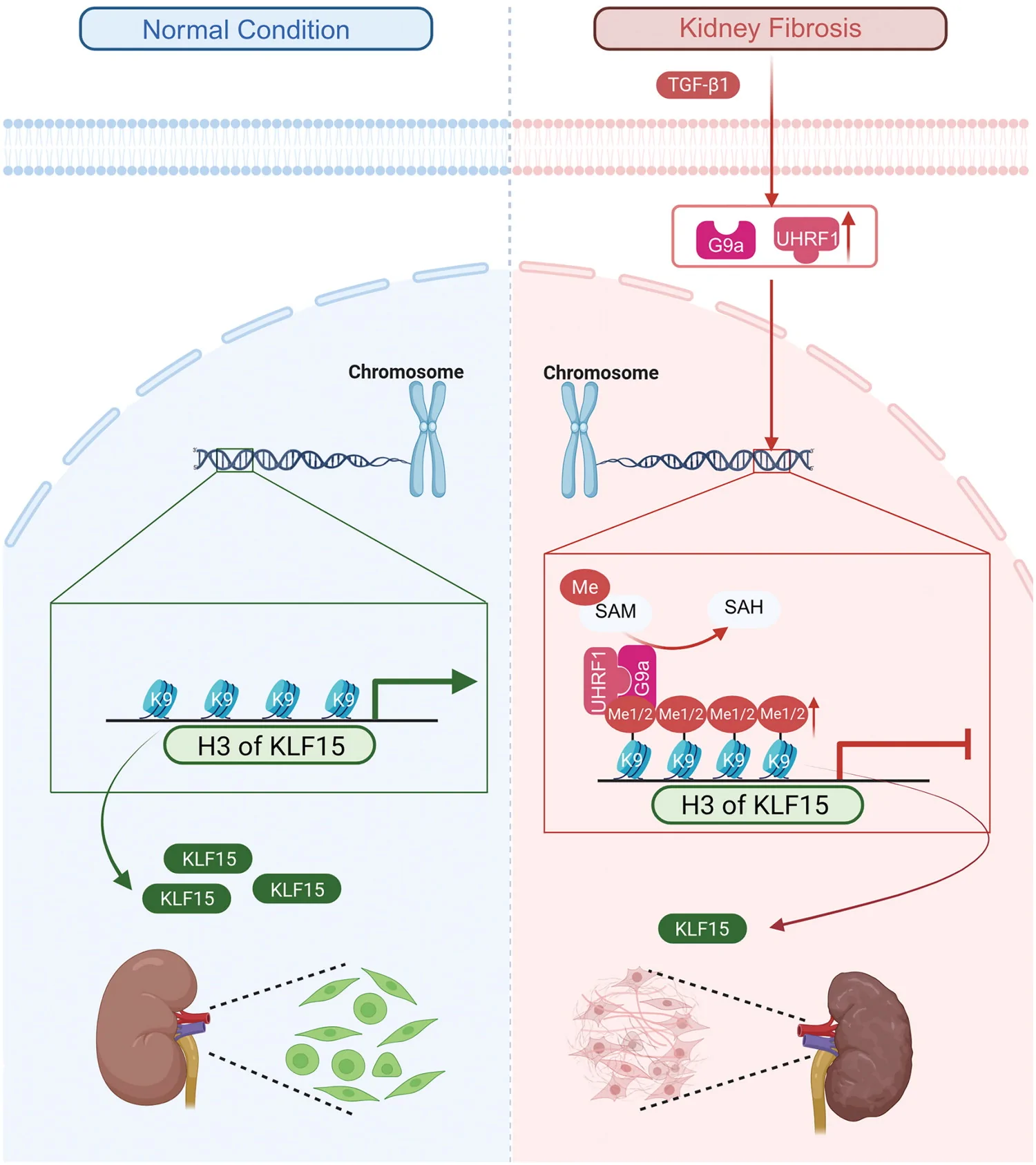
Schematic model depicting the proposed mechanism of the UHRF1/G9a axis in renal fibrosis. In response to TGF-β1 stimulation, UHRF1 upregulates G9a expression and enhance its enzymatic activity, which catalyzes the deposition of H3K9me1 and H3K9me2 at the promoter of the anti-fibrotic gene *KLF15*, leading to its transcriptional repression and the exacerbation of renal interstitial fibrosis.

Taken together, this study demonstrates that downregulation of UHRF1 expression inhibits the expression and enzymatic activity of G9a. This suppression reduces the enrichment of H3K9me1 and H3K9me2 at the promoter of *KLF15*, thereby preserving KLF15 expression and may be associated with the alleviation of renal fibrosis (Figure 7).

Although CKD arises from diverse etiologies, renal fibrosis represents a common pathological endpoint, with interstitial fibrosis serving as a key morphological hallmark [36]. Activated fibroblasts are well-established as central effector cells in fibrosis across multiple organs, driving excessive deposition of ECM proteins [37]. Therefore, therapeutic strategies targeting fibroblast activation remain a major focus in anti-fibrotic research.

Studies have reported that UHRF1 is highly expressed in rapidly proliferating cells [38] and promotes the progression of various diseases, including myeloid leukemia [39], pancreatic cancer, gastric cancer [40], and intrahepatic cholangiocarcinoma [41]. In the injured kidney, renal tubular epithelial cells exhibit a relatively slow and often incomplete repair process owing to their intrinsic cellular properties [42]. In contrast, myofibroblasts are vigorously activated and undergo rapid proliferation, serving as the principal effector cells that propel fibrotic progression [43]. Given the characteristic of UHRF1 being higher expressed in renal fibroblasts, we ultimately chose fibroblasts as our *in vitro* experimental model. Previous research has predominantly focused on its role in regulating DNA methylation. Our group’s previous studies further demonstrated that UHRF1 recruits DNMTs to promote DNA methylation of anti-fibrotic gene *KLF15*, thereby contributing to renal fibrosis [7]. However, it’s reported that UHRF1 functions as more than a simple DNA methylation regulator but also as a scaffold protein. Emerging evidence indicates that UHRF1 can simultaneously recruit multiple epigenetic modifiers *via* its SRA and RING domains, including G9a, histone deacetylase 1 (HDAC1), lysine acetyltransferase 5, and DNA methyltransferases (DNMTs) to form a complex. This scaffold property positions UHRF1 as a central hub in epigenetic regulation [18,44,45]. For example, UHRF1 has been shown to recruit the histone methyltransferase G9a to the BRCA1 promoter, facilitating H3K9 methylation [46]. However, reports on the role of UHRF1-G9a and downstream histone methylation modifications in renal fibrosis remain insufficient.

In this study, we demonstrate that UHRF1 functionally interacts with G9a through its SRA and RING domains and mediates both the expression and enzymatic activity of G9a in the regulation of histone methylation modifications. Together, UHRF1-G9a drive increased H3K9me1 and H3K9me2 deposition at the promoter of *KLF15*, which is associated with its transcriptional repression. KLF15 thus emerges as one of the key downstream candidates through which the UHRF1-G9a axis may promote renal fibrosis. We first observed that UHRF1 and G9a were concomitantly upregulated in renal fibrosis models. Notably, the elevated expression and enzymatic activity of G9a in these models were partially reversed upon *UHRF1* knockdown *in vitro* or fibroblast-specific knockout *in vivo*. However, modulating G9a expression or activity did not significantly alter UHRF1 expression, indicating that G9a operates downstream of UHRF1. Our present findings further extend the current understanding of UHRF1 as an epigenetic hub in renal fibrosis, uncovering that UHRF1 promotes fibrogenesis not only through DNA methylation but also by mediating G9aexpression or activity to drive histone methylation. The inhibition of UHRF1 could simultaneously disrupt these two major epigenetic pathways, suggesting an important regulatory role of UHRF1 in renal fibrosis.

Furthermore, inhibition of G9a expression or activity alone alleviated renal fibrosis independently of UHRF1, in both cellular and animal models. We attribute this effect to G9a-mediated enrichment of H3K9me1/me2 *via* its intrinsic methyltransferase activity, which mediates H3K9me1/me2 deposition at the *KLF15* promoter region and leads to transcriptional repression of *KLF15*. However, emerging evidence indicates that G9a not only mediates histone methylation but also contributes to DNA methylation in a manner similar to UHRF1, suggesting possible functional overlap between the two proteins [47,48]. Moreover, G9a is known to methylate a range of non-histone substrates [49]. Thus, the precise role of G9a in renal fibrosis merits further mechanistic investigation.

KLF15 is a well-documented anti-fibrotic transcription factor in multiple organs, including the liver, heart and lung [50–53], with a particularly well-recognized role in the kidney [7,11, 54,55]. Based on public database screening and literature review, we hypothesized that KLF15 might be a promising candidate in the UHRF1/G9a-mediated fibrotic pathway and this was subsequently validated by experiments. As demonstrated in our study, the epigenetic silencing of *KLF15* may be associated with the progression of renal fibrosis. However, given the complexity of the anti-fibrotic effects observed upon UHRF1-G9a inhibition, we acknowledge that the downstream regulatory targets of the UHRF1-G9a axis may extend well beyond *KLF15*, and additional downstream genes remain to be identified in future studies. This suggests that UHRF1-G9a likely promotes fibrotic progression through a broader transcriptional repression network, which warrants systematic mapping in future studies.

This study has several limitations. First, G9a is involved not only in histone methylation but also in DNA methylation, a function analogous to that of UHRF1, suggesting potential overlap between the two proteins [48,56]. The contribution of G9a-mediated DNA methylation to renal fibrosis, however, remains unexplored. Second, while the SRA and RING domains of UHRF1 are primarily responsible for its direct interaction with G9a [19] and were therefore the focus of our functional assays, the roles of its PHD and TTD domains, which are known to recognize histone marks for chromatin recruitment [57], in regulating G9a activity and fibrosis remain to be determined. Third, our *in vivo* validation relied on systemic pharmacological inhibition of G9a rather than kidney fibroblast-specific genetic knockout, which may limit the cellular resolution and specificity of the conclusions drawn. Fourth, this study focused on fibroblasts and did not examine whether the UHRF1-G9a-KLF15 axis also operates in other renal cell types, such as tubular epithelial cells, which are known to play critical roles in renal fibrosis *via* incomplete repair and profibrotic cytokine secretion [58,59]. Finally, while our study demonstrates the involvement of the UHRF1-G9a axis in renal fibrosis, no inhibitors of UHRF1 or G9a have yet entered clinical trials. Whether the UHRF1‑G9a axis confers renoprotective effects and delays CKD progression remains uncertain based on our current data.

In summary, this study delineates an important epigenetic axis in renal fibrosis, wherein UHRF1 interacts with G9a through its SRA and RING domains. *KLF15* is identified as one of the downstream candidates of this axis. UHRF1-G9a-mediated deposition of repressive H3K9me1/me2 marks at the *KLF15* promoter leads to its transcriptional silencing, which is associated with fibroblast activation and ECM accumulation. This mechanism was consistently validated in patient tissues, multiple murine fibrosis models, and TGF-β1-stimulated renal fibroblasts. Our work not only reveals the appreciable role of the UHRF1/G9a axis in renal fibrogenesis but also provides a novel theoretical foundation for the involvement of epigenetic regulation in renal fibrosis.

## Conclusions

This work defines a key pathogenic epigenetic circuit in renal fibrosis centered on the UHRF1/G9a axis. We demonstrate that UHRF1 affects the expression and activity of G9a, UHRF1-G9a mediates H3K9me1/me2-dependent silencing of *KLF15*, which is associated with fibroblast activation and matrix deposition in the context of renal fibrosis. Importantly, inhibition at multiple nodes of this axis, including UHRF1, G9a, or their interaction, may effectively ameliorate fibrosis. Our results refine the theoretical framework that UHRF1 is a master regulator that integrates multiple repressive chromatin modifications and highlight the potential of disrupting the UHRF1/G9a complex to alleviate renal fibrosis.

## Ethics approval and consent to participate

All human data involved in this study were obtained with written informed consent (including consent for the publication of the data), in accordance with the ethical approvals from the Ethics Committee of Zhongshan Hospital, Fudan University (Approval No. B2021-346R) and the 1964 Helsinki Declaration. All animal experiments complied with the ethical guidelines of the Animal Center of Fudan University (Approval number: 202312008S).

## Supplementary Material

Supplementary_figure_5_01.tif

Supplementary_figure_3_01.tif

Supplementary_figure_9_01.tif

Supplementary_figure_1_01.tif

Supplementary_figure_8_01.tif

Supplementary table S2.docx

Supplementary_figure_2_01.tif

Supplementary_figure_6_01.tif

Supplementary table S1.docx

Supplementary_figure_4_01.tif

Supplementary_figure_7_01.tif

Supplementary Material and Methods_cleaned.docx

## Data statement and availability

Data for Figures 1A and 6A were generated from the public RNA-seq datasets, which are available in the GEO database (https://www.ncbi.nlm.nih.gov/geo/) under accession code GSE66494, GSE269356, GSE299417 and GSE293589 respectively. Data for Figures 4A and S^6^A, B were generated using the software GRAMM-X (http://gramm.compbio.ku.edu/) and evaluated by software GROMACS (2025.3). Supporting information is available from the corresponding author upon reasonable request.
