# Supplementary material for: Exacerbation of renal interstitial fibrosis by the UHRF1/G9a axis through epigenetic silencing of KLF15
Source: Ren Fail. 2026 Sep 21;48(1):2719260. doi: 10.1080/0886022X.2026.2719260 (PMC13600313; doi:10.1080/0886022X.2026.2719260)
Supplement: Supplementary file 1 [file IRNF_A_2719260_SM6556.docx]

**Supplementary Material and Methods**

**Human Renal Biopsy Samples**

Human renal biopsy samples were acquired as part of routine clinical diagnostics from patients admitted to the department of Nephrology, Zhongshan Hospital, Fudan University between June 2021 and December 2022 (clinical details are summarized in Supplementary Table S1). All participants provided written informed consent, and the study protocol was approved by the Ethics Committee of Zhongshan Hospital, Fudan University (Approval No. B2021-346R). The renal tissue obtained via renal biopsy was immediately cryopreserved, followed by fixation in 4% paraformaldehyde overnight, paraffin embedding, and sectioning into 4μm-thick sections for subsequent histopathological staining.

**Animals and treatment**

Wild-type male C57/BL6 mice aged 6-8 weeks (purchased from Shanghai Model Organisms Center, Inc.) were housed in the Experimental Animal Center of Fenglin Campus, Fudan University, under a 12-hour light -dark cycle with free access to food and water. All animals were acclimated to this environment for 1 week before the experiment. After acclimation, the mice were randomly grouped according to the cage positions, and underwent unilateral ureteral obstruction (UUO) or unilateral renal ischemia-reperfusion injury (UIR) surgery to induce renal fibrosis. Anesthesia was performed with freshly prepared 2% sodium pentobarbital solution administered via intraperitoneal injection at a dose of 50 mg/kg body weight. When the mice exhibited complete muscle relaxation and loss of the righting reflex, hair removal and surgery were subsequently conducted. For the UUO surgery, the left ureter was ligated with 5-0 silk thread. For the UIR surgery, the left renal pedicle was clamped with a microaneurysm clip for 40 minutes while maintaining the body temperature at 38 °C. In the sham-operated group, only the left kidney was exposed without any treatment. To investigate the effect of the G9a inhibitor BIX-01294 on renal fibrosis, starting from the day of modeling until the day before kidney collection, the two fibrosis model mice were intraperitoneally injected daily with BIX-01294 (MedChemExpress, HY-10587) dissolved in DMSO and subsequently diluted with normal saline (working solution concentration: 1 mg/mL). The experimental group was intraperitoneally administered BIX-01294 at 2 mg/kg, while the control group received an equal volume of normal saline. Kidney samples were collected one week after UUO and two weeks after UIR for protein analysis and histological examination. In all animal experiments, mice were euthanized by cervical dislocation performed by trained personnel at the experimental endpoint in accordance with the American Veterinary Medical Association (AVMA) guidelines. The animal experiment protocol has been reviewed and approved by the Institutional Animal Care and Use Committee of Fudan University (Approval number: 202312008S). Our study only focused on male mice, and it is currently unclear whether these findings are applicable to female mice. In future experiments, we will further verify the role of our research in female mice.

**Construction of Fibroblast-Specific UHRF1 Conditional Knockout Mice**

UHRF1^flox/flox^ mice and Col1a2-Cre/ERT2 transgenic mice were purchased from the Shanghai Model Organisms Center. To generate an inducible fibroblast-specific UHRF1 conditional knockout mouse model, the UHRF1^flox/flox^ mice were crossed with Col1a2-Cre/ERT2 transgenic mice. The Cre-ERT system was induced by intraperitoneal injection of 100 mg/kg tamoxifen (MedChemExpress, HY-13757A) for 5 consecutive days, starting one week prior to surgery in homozygous male mice (Col1a2-Cre⁺UHRF1^flox/flox^). After UUO or UIR surgeries, tamoxifen was continuously administered daily until the day before sacrifice.

**Renal Pathological Staining**

Mouse kidneys were fixed in 4% paraformaldehyde, embedded in paraffin, and cut into 3 μm-thick sections for performing Masson’s trichrome staining, immunohistochemistry and immunofluorescence. The kidney sections were first dewaxed and then subjected to antigen retrieval in heated Tris-EDTA buffer. Subsequently, the sections were incubated in 3% hydrogen peroxide for 10 minutes to block endogenous peroxidase activity and 5% bovine serum albumin (BSA) for 1 hour for blocking treatment. The primary antibodies used in this study were anti-α-smooth muscle actin (α-SMA, 1:200, 67735-1-IG, Proteintech), anti-fibronectin (1:200, T59537, Abmart), anti-UHRF1 (1:100, sc-373750, Santa Cruz Biotechnology), anti-G9a (1:200, ab185050, Abcam), anti-SETDB1 (1:200, 11231-1-AP, Proteintech), anti-H3K9me1 (1:500, ab176880, Abcam), anti-H3K9me2 (1:200, ab176882, Abcam) and anti-H3K9me3 (1:200, 61013, Proteintech). The secondary antibody incubation and diaminobenzidine (DAB) staining were carried out using the GTVision Ⅲ Detection System kit (Gene Tech, Shanghai, China). Images were captured using an optical microscope. Ten random fields were selected from each sample and analyzed using Image J software.

**Immunofluorescence**

Mouse kidneys were fixed in 4% paraformaldehyde, embedded in paraffin, and cut into 3 μm-thick sections. After dewaxing and antigen retrieval, the sections were incubated in 3% hydrogen peroxide for 10 minutes to block endogenous peroxidase activity and 5% BSA for 1 hour for blocking treatment. The primary antibodies used in this study were anti-UHRF1, anti-G9a and anti-platelet-derived growth factor receptor α+β (PDGFRα+β, 1:200, ab32570, Abcam, AQP1, 1:200, ab168387, Abcam; UHRF1, 1:100, sc-373750, Santa Cruz Biotechnology; G9a, 1:200, ab185050, Abcam). The cell nuclei were stained with DAPI. Images were captured using an Olympus FV3000 confocal microscope.

**Cell Culture and Treatment**

Rat normal renal fibroblasts (NRK-49F cells, Yizefeng Biotechnology) were cultured in high glucose Dulbecco's Modified Eagle Medium (DMEM, KGL1206-500, KeyGENE Biotechnology) supplemented with 10% fetal bovine serum (FBS, 12103C, Sigma) and 1% penicillin and streptomycin. Before any stimulation, NRK-49F cells were starved overnight in DMEM medium containing 0% FBS. Based on the results of time and concentration gradients, Transforming Growth Factor-β1 (TGF-β1, 7754-BH, R&D) was used at a concentration of 10 ng/mL for 48 hours. Based on the results of CCK-8 assay (Supplementary Figure 4), the concentration of 2.5 μM was selected, as it effectively reduced the expression of fibrotic markers in fibroblasts without compromising cell viability (Supplementary Figure 4, Figure 5G). Because BIX-01294 was added simultaneously with TGF-β1 for intervention, we selected a treatment duration of 48 hours.

**In Vitro Genes Transfection**

NRK-49F cells were seeded in 12-well culture dishes. For the small interfering RNA (siRNA) transfection, when the cells reached 70-80% confluence the next day, the siRNA or the negative control siRNA was transfected into the cells using Lipofectamine 3000 according to the manufacturer's instructions (L3000150, Invitrogen Life Technologies). The sequence of siRNA used for rat *UHRF1* interference was as follows: the sense strand was 5’ - GAGGAGCTGTTTCACGTGGAA(dT)(dT) - 3’ and the antisense strand was 5’ - UUCCACGTGAAACAGCUCCTC(dT)(dT) - 3’. The sequence of siRNA used for rat *G9a* interference was as follows: the sense strand was 5’ - UUCUCCGAACGUGUCACGU - 3’ and the antisense strand was 5’ - ACGUGACACGUUCGGAGAA - 3’. For lentiviral transduction, cells at 50% confluence were transduced with lentivirus encoding rat *UHRF1* SRA/RING truncation mutant (deleting amino acids 416-578 and 705-744) or the full-length vector control, in the presence of polybrene as per the manufacturer's instructions (C0351, Beyotime). For the overexpression plasmid transfection, when the cells reached 70-80% confluence the next day, the plasmid or the negative control plasmid was transfected into the cells using Lipofectamine 3000 according to the manufacturer's instructions. The sequence of rat *KLF15* overexpression plasmid was as follows: the forward sequence was 5’ - CACCAGTGGGGGTGGTACCC - 3’ and the reverse sequence was 5’ - GAGCGTATGTTAGTACTATCG - 3’. 8 hours later, the cells were washed and cultured overnight in fresh serum-free DMEM medium, and then the cells were stimulated with 10 ng/mL of TGF-β1 for 48 hours.

**Western Blot Analysis**

Kidney tissues or cells were lysed in radioimmunoprecipitation assay (RIPA) lysis buffer containing protease and phosphatase inhibitor cocktail. The protein was separated by SDS-PAGE, and transferred to PVDF membranes. Blots were probed with primary antibodies against UHRF1 (1:500, sc-373750, Santa Cruz Biotechnology), G9a (1:1000, ab185050, Abcam), Fibronectin (1:2000, T59537, Abmart), α-SMA (1:1000, 67735-1-IG, Proteintech), KLF15 (ab167192, Abcam), GAPDH (1:10000, 10494-1-AP, Proteintech), H3K9me1 (1:10000, ab176880, Abcam), H3K9me2 (1:1000, ab176882, Abcam), H3K9me3 (1:1000, 61013, Proteintech) and H3 (1:1000, 17168-1-AP, Proteintech). The intensity of the bands was quantitatively analyzed using Image J software.

**Measurement of serum creatinine and blood urea nitrogen**

Before euthanasia, blood samples were obtained via retro-orbital bleeding. Serum was isolated from blood samples after a 60-min clotting period at room temperature, followed by centrifugation at 2000 xg for 10 min. The clarified, yellowish supernatant (serum) was collected for subsequent analysis. Serum creatinine and blood urea nitrogen (BUN) levels were measured using commercially available assay kits (DICT-500 and DIUR-500, BioAssay Systems) according to the manufacturer’ protocols. The blood creatinine level was measured at an absorbance of OD570nm, and the blood urea nitrogen level was measured at an absorbance of OD520nm after incubation at 37℃ for 30 min.

**G9a Enzymatic Activity Measurements**

NRK - 49F cells were seeded in 12-well culture dishes. When the cells reached 70-80% confluence the next day, the N-terminal GFP-tagged G9a overexpression plasmid was transfected into the cells using Lipofectamine 3000 according to the manufacturer's instructions (L3000150, Invitrogen Life Technologies). The sequence of rat G9a overexpression plasmid was as follows: the forward sequence was 5’ - CACACTGGACTAGTGGATCCCGCCACCATGCGGGGTCTGCCGAGAGGGAGGGGGCTGATGCGG - 3’ and the reverse sequence was 5’ - AGTCACTTAAGCTTGGTACCGAGGTGTTGATGGGGGGCAGGGAGCTGAG - 3’. 8 hours later, the cells were washed and cultured in fresh complete medium, followed by cell incubation for subsequent experimental interventions. After lysing the cells with RIPA lysis buffer containing protease and phosphatase inhibitors cocktails, the lysates were incubated with Anti-GFP Magnetic Beads (1:10, P2132, Beyotime) at 4℃ overnight. Subsequently, competitive elution was performed by elution buffer (21004, Thermo Fisher). The samples were titrated to normalize the concentrations of GFP-G9a complexes. For the detection of G9a activity in kidney tissue, it is essential to use fresh kidney samples. Tissues should be thoroughly homogenized and lysed, and 50 mg of kidney tissue per sample should be used to obtain relatively enriched G9a protein. The remaining steps are the same as those for measuring enzymatic activity in cell-based experiments. Protein quantification was performed using a bicinchoninic acid (BCA) protein assay kit. Subsequently, G9a activity was assayed using the G9a enzymic activity Kit (52051, BioScience). After incubation at 37 °C for 4 hours, the absorbance was measured at a wavelength of 560 nm. Net absorbance was normalized to total protein concentration to obtain specific activity, which was then expressed as fold change relative to the control group.

**Co-Immunoprecipitation (Co - IP)**

Cells seeded in 10cm culture dishes were collected into immunoprecipitation lysis buffer containing protease and phosphatase inhibitor cocktail. Immunoprecipitation was carried out using anti-UHRF1 (1:100, 21401-1-AP, Proteintech; recognizing N-terminal residues 1–200), anti-IgG (1:20, 3900, Cell Signaling Technology) and protein A/G magnetic beads according to the manufacturer's protocol (1:10, P2179, Beyotime). The captured antigen-antibody complexes were eluted with 2×sodium dodecyl sulfate (SDS) buffer and analyzed by Western blotting. IgG was used as a negative control.

**Cell Counting Kit-8 (CCK - 8) Assay**

The CCK-8 assay was performed to evaluate the cytotoxic effect of BIX-01294 on NRK-49F cells (Fig. S4). Cells were seeded at 3,000 per well in 96-well plates and cultured until reaching 70-80% confluence. Subsequently, the cells were treated with 10 ng/mL TGF-β1, DMSO (solvent control) and BIX-01294 at concentrations of 2.5 μM, 5 μM, 10 μM, 20 μM for 48 hours. Wash the wells and added 100 μL fresh DMEM and 10 μL CCK-8 reagent per well. The plate was incubated at 37 ℃ for 2 hours, followed by measurement of absorbance at a wavelength of 450 nm.

**Chromatin Immunoprecipitation (ChIP)-Quantitative Polymerase Chain Reaction (qPCR)**

1 × 10^7^ NRK-49F cells were taken from each sample, crosslinked with the methanol-free 1% formaldehyde at room temperature for 15 minutes, and then the chromatin immunoprecipitation experiment was carried out using a chromatin immunoprecipitation kit (RK20258, Abclonal). Subsequently, the chromatin was fragmented into fragments approximately 300bp in length by sonication. Then, the fragmented chromatin was incubated overnight with specific primary antibodies targeting H3 (1:100, ab1791, Abcam), H3K9me1 (1:100, ab176880, Abcam) and H3K9me2 (1:50, ab176882, Abcam) at 4 °C overnight. ChIP protein G magnetic beads (1:10, P2089, Beyotime) were incubated with rotation for 3 hours. Subsequently, the microbeads were washed successively with washing buffers of different salt concentrations and finally eluted with an elution buffer containing 1% sodium dodecyl sulfate (SDS). The obtained antibody-chromatin complexes were subjected to reverse crosslinking and DNA purification steps (69504, QIAGEN) in sequence. The obtained DNA samples were subjected to real-time quantitative polymerase chain reaction (RT-qPCR) using the following primers. The forward primer: 5’ - CCCGTGAGTAACCATGACCG - 3’ and reverse primer: 5’ - GAATCCACTGAAAGCCCGCA - 3’. The primers were used to determine the H3K9me1/me2 enrichment degree of *KLF15* promoter region. And the ChIP-qPCR signals were first normalized to the corresponding input DNA, then further normalized to histone H3 levels at the same region to exclude the potential confounding effect of altered nucleosome density upon UHRF1 deletion.

**Cleavage Under Targets and Tagmentation (CUT&Tag)**

CUT&Tag libraries were constructed using a Hyperactive Kit according to the manufacturer's instructions (TD904, Vazyme). 1 × 10⁵ formaldehyde-crosslinked cells were harvested and washed twice with ice-cold PBS, resuspend cells in antibody binding buffer with primary antibody targeting the protein of H3K9me1 (1μg, ab176880, Abcam) and H3K9me2 (1μg, ab176882, Abcam), incubated at 4 °C for 1 h with gentle rotation. Added pA-Tn5 transposase adapter complex (TD202, Vazyme) in each sample and incubated at room temperature for 1 h to allow targeted binding of the complex to the antibody-antigen conjugate. Subsequently added Mg²^+^-containing activation buffer to initiate in situ tagmentation reaction at 37 °C for 1 h and terminated the reaction with EDTA-containing stop buffer. Extracted genomic DNA using DNA extraction kit (69504, QIAGEN), amplified the tagmented DNA fragments with index-containing PCR primers for 12 cycles and purified the PCR products with VAHTS DNA clean beads (N411-01, Vazyme). Finally, performed high-throughput sequencing on the Illumina HiSeq 6000 platform. Enrichment of H3K9me1/me2 at the *KLF15* promoter was visualized using the Integrative Genomics Viewer software.

**Molecular Docking and Molecular Dynamics Simulation**

Protein corresponding crystal structures of UHRF1 and G9a were obtained from the Uniprot website (https://www.uniprot.org/) and were preprocessed (including dehydration, residue supplementation, and structural correction) by the PDB Prep Server website (https://swift.cmbi.umcn.nl/servers/html/model.html). Docking was performed via GRAMM-X (http://gramm.compbio.ku.edu/), and binding energies were evaluated by GROMACS software (2025.3). Prediction of the binding domains between UHRF1 and G9a was based on CD-search website(https://www.ncbi.nlm.nih.gov/Structure/cdd/wrpsb.cgi).

**Bioinformatics Analysis**

For datasets with raw count data, differential expression analysis was performed using the DESeq2 package (v1.34.0) in R (v4.1.0). For datasets with FPKM-normalized expression values, differential expression analysis was performed using the limma package (v3.50.0) with the voom function. Genes with an |log_2_FC| > 1 and an adjusted.*P* < 0.05 were considered significantly differentially expressed. Volcano plots were generated using the ggplot2 package (v3.4.0) in R (v4.1.0) to visualize the distribution of differentially expressed genes (DEGs).

**Statistical Analysis**

All data are presented as mean ± standard deviation. For comparisons between two groups, an unpaired two-tailed Student’s t-test was used. For comparisons involving a single independent variable with three or more groups, one-way analysis of variance (ANOVA) was performed, followed by Tukey’s post hoc test for multiple comparisons. For multiple comparisons involving two independent variables, two-way ANOVA was used, followed by Šidák’s post hoc test as appropriate. All statistical tests were two-tailed, and a P.value < 0.05 was considered statistically significant. Analyses were conducted using GraphPad Prism version 9.1.1.
