# Supplementary material for: Exacerbation of renal interstitial fibrosis by the UHRF1/G9a axis through epigenetic silencing of KLF15
Source: Ren Fail. 2026 Sep 21;48(1):2719260. doi: 10.1080/0886022X.2026.2719260 (PMC13600313; doi:10.1080/0886022X.2026.2719260)
Supplement: Supplementary file 2 [file IRNF_A_2719260_SM6559.docx]

|  | CKD 1 | CKD 2 | CKD 3a | CKD 3b | CKD 4 | CKD 5 | *P* |
| --- | --- | --- | --- | --- | --- | --- | --- |
|  | (n=6) | (n=6) | (n=6) | (n=6) | (n=6) | (n=6) |  |
| Sex (male) | 3 (50.0%) | 3 (50.0%) | 4 (60.0%) | 5 (83.3%) | 4 (60.0%) | 3 (50.0%) | 0.450 |
| Age (years) | 41.1±16.5 | 44.8±14.1 | 45.7±14.3 | 60.3±9.5 | 53.3±12.9 | 55.8±16.5 | 0.074 |
| SBP (mmHg) | 137.8±22.8 | 128.0±32.0 | 146.3±35.1 | 143.8±28.6 | 167.7±38.5 | 196.3±47.8 | 0.007 |
| DBP (mmHg) | 94.8±11.5 | 94.2±20.8 | 87.5±17.7 | 90.2±17.3 | 101.0±15.7 | 106.7±22.6 | 0.158 |
| Hb (g/L) | 139.5±20.2 | 129.2±8.3 | 116.5±31.2 | 122.7±16.0 | 112.7±15.7 | 108.5±9.5 | 0.105 |
| FPG (mmol/L) | 5.1±0.8 | 4.7±0.5 | 5.1±0.6 | 6.7±2.2 | 5.3±1.1 | 6.8±3.2 | 0.003 |
| HbA1c (%) | 5.9±0.6 | 5.3±0.6 | 5.5±0.4 | 5.8±0.6 | 5.7±0.8 | 6.0±2.0 | 0.391 |
| Serum albumin (g/L) | 36.7±7.9 | 39.3±7.6 | 39.8±9.2 | 37.8±8.4 | 38.8±5.5 | 32.3±8.7 | 0.248 |
| TG (mmol/L) | 2.5±1.4 | 2.2±1.0 | 1.7±0.8 | 3.9±1.8 | 2.6±1.8 | 2.2±1.4 | 0.032 |
| TC (mmol/L) | 6.1±3.0 | 4.1±0.9 | 6.8±3.7 | 5.3±1.0 | 3.9±1.3 | 5.9±3.1 | 0.400 |
| SCr (μmol/L) | 72.2±13.9 | 101.8±15.1 | 130.8±13.5 | 183.8±29.1 | 298.7±60.6 | 622.3±243.0 | <0.001 |
| SUA (μmol/L) | 314.7±132.9 | 391.0±196.1 | 380.3±103.5 | 390.5±107.6 | 516.5±170.3 | 409.3±124.2 | 0.226 |
| BUN (mmol/L) | 5.6±1.6 | 6.5±1.8 | 9.7±4.9 | 9.4±2.7 | 16.1±3.3 | 25.7±10.2 | <0.001 |
| eGFR (mL/min/1.73 m^2^) | 104.9±12.0 | 68.5±6.8 | 50.1±7.7 | 32.0±5.5 | 18.1±5.1 | 8.5±4.1 | <0.001 |

**Supplementary Table S1. Clinical characteristics of the participants enrolled in this study**
