# Supplementary material for: Exacerbation of renal interstitial fibrosis by the UHRF1/G9a axis through epigenetic silencing of KLF15
Source: Ren Fail. 2026 Sep 21;48(1):2719260. doi: 10.1080/0886022X.2026.2719260 (PMC13600313; doi:10.1080/0886022X.2026.2719260)
Supplement: Supplementary file 3 [file IRNF_A_2719260_SM6562.docx]

|  | **UHRF1** | **G9a** | **Distance (Å)** |
| --- | --- | --- | --- |
| **hydrogen bond**  **(human)** | ARG6 | ASP1209 | 1.5 |
|  | LYS11 | ASN1060 | 3.2 |
|  | THR13 | LYS11 | 1.6 |
|  | THR13 | ASP1206 | 2.6 |
|  | ASN17 | GLU1226 | 3.6 |
|  | THR413 | GLU414 | 2.8 |
|  | THR413 | SER1240 | 3.4 |
|  | LYS644 | GLU154 | 3.1 |
|  | GLN647 | GLU149 | 3.3 |
|  | ASP1206 | LYS11 | 2.0 |
|  | GLY1208 | THR13 | 2.8 |
|  | LYS1227 | THR13 | 3.3 |
|  | LYS1227 | ASN621 | 1.5 |
|  | GLN1239 | GLN712 | 2.7 |
|  | GLN1239 | SER1240 | 2.4 |
| **hydrogen bond**  **(mouse)** | MET8 | ASP9 | 2.9 |
|  | ARG36 | HIS38 | 3.2 |
|  | GLU62 | LYS288 | 2.5 |
|  | VAL73 | GLU835 | 3.0 |
|  | GLN75 | GLU835 | 3.1 |
|  | GLN75 | ARG873 | 3.4 |
|  | SER82 | ARG641 | 1.8 |
|  | SER82 | ILE922 | 2.4 |
|  | SER82 | CYS937 | 1.8 |
|  | THR83 | ARG641 | 3.2 |
|  | SER118 | SER119 | 2.5 |
|  | SER119 | ASP585 | 3.2 |
|  | SER119 | ARG733 | 1.5 |
|  | SER125 | LEU766 | 2.8 |
|  | GLU307 | ARG763 | 3.0 |
|  | GLU310 | ARG763 | 3.6 |
|  | GLU318 | ARG763 | 3.2 |
|  | GLU318 | LEU766 | 3.1 |
|  | LYS383 | ASP284 | 2.1 |
|  | SER388 | ASP286 | 3.3 |
|  | ARG641 | HIS38 | 3.2 |
|  | ARG641 | THR642 | 3.2 |
|  | LYS688 | GLU630 | 1.6 |
|  | LYS688 | GLN664 | 3.0 |
|  | GLN740 | LYS688 | 3.0 |
|  | LEU766 | GLU318 | 3.1 |
|  | ARG768 | GLU310 | 3.2 |
| **hydrogen bond**  **(rat)** | GLU189 | HIS190 | 2.3 |
|  | PRO297 | ARG392 | 1.7 |
|  | SER298 | GLN300 | 2.4 |
|  | THR347 | GLU378 | 2.8 |
|  | SER389 | GLN343 | 2.5 |
|  | LYS394 | GLN300 | 2.1 |
|  | SER403 | LYS407 | 2.4 |
|  | PRO404 | ARG48 | 1.9 |
|  | TRP405 | ASP363 | 3.4 |
|  | THR642 | GLY643 | 2.4 |
|  | SER652 | LYS638 | 3.3 |
|  | LYS654 | ALA617 | 3.3 |
|  | GLU701 | THR68 | 2.3 |

**Supplementary Table S2. The amino acid sites and distances of the interaction between protein UHRF1 and protein G9a in human, mouse and rat**
